# “Point of no return” in unilateral renal ischemia reperfusion injury in mice

**DOI:** 10.1186/s12929-020-0623-9

**Published:** 2020-02-14

**Authors:** Alexander Holderied, Franziska Kraft, Julian Aurelio Marschner, Marc Weidenbusch, Hans-Joachim Anders

**Affiliations:** 1 Department of Nephrology and Medical Intensive Care, Charité, Universitätsmedizin Berlin, Berlin, Germany; 2 Medizinische Klinik and Poliklinik IV, Klinikum der Universität München, Munich, Germany

**Keywords:** Ischemia/reperfusion injury, Chronic on acute kidney injury, Acute tubule necrosis, Necroinflammation, Renal scarring

## Abstract

**Background:**

In the past years evidence has been growing about the interconnection of chronic kidney disease and acute kidney injury. The underlying pathophysiological mechanisms remain unclear. We hypothesized, that a threshold ischemia time in unilateral ischemia/reperfusion injury sets an extent of ischemic tubule necrosis, which as “point of no return” leads to progressive injury. This progress is temporarily associated by increased markers of inflammation and results in fibrosis and atrophy of the ischemic kidney.

**Methods:**

Acute tubule necrosis was induced by unilateral ischemia/reperfusion injury in male C57BL/6 N mice with different ischemia times (15, 25, 35, and 45 min). At multiple time points between 15 min and 5 weeks we assessed gene expression of markers for injury, inflammation, and fibrosis, histologically the injury of tubules, cell death (TUNEL), macrophages, neutrophil influx and kidney atrophy.

**Results:**

Unilateral ischemia for 15 and 25 min induced upregulation of markers for injury after reperfusion for 24 h but no upregulation after 5 weeks. None of the markers for inflammation or fibrosis were upregulated after ischemia for 15 and 25 min at 24 h or 5 weeks on a gene expression level, except for *Il-6*. Ischemia for 35 and 45 min consistently induced upregulation of markers for inflammation, injury, and partially of fibrosis (*Tgf-β1* and *Col1a1*) at 24 h and 5 weeks. The threshold ischemia time for persistent injury of 35 min induced a temporal association of markers for inflammation and injury with peaks between 6 h and 7 d along the course of 10 d. This ischemia time also induced persistent cell death (TUNEL) throughout observation for 5 weeks with a peak at 6 h and progressing kidney atrophy beginning 7 d after ischemia.

**Conclusions:**

This study confirms the evidence of a threshold extent of ischemic injury in which markers of injury, inflammation and fibrosis do not decline to baseline but remain upregulated assessed in long term outcome (5 weeks). Excess of this threshold as “point of no return” leads to persistent cell death and progressing atrophy and is characterized by a temporal association of markers for inflammation and injury.

## Background

Recovery of excretory function after acute tubule necrosis (ATN) is common, hence it has been considered a transient disease. However, it has become evident that ATN is frequently followed by chronic kidney disease (CKD). After survival of an episode of ATN the long term outcome and development of CKD is of major importance as among others this determines cardiovascular events and risk for end-stage renal disease [1–5].

Several mechanisms have been described for the transition from ATN to CKD. Some studies have found fibrosis to not only play a role as consequence of insufficient repair, but as self-perpetuating mechanism of progression to CKD [6]. The maladaptive repair in which dedifferentiated tubule epithelial cells cannot proliferate due to a G2-M phase arrest as leading mechanism is one discussed mechanism [7]. Another study indicated, that the capacity of the kidney to restore its function after ATN mainly is a consequence of remnant tubule epithelial cell hypertrophy and limited tubule epithelial cell division by specific renal progenitor cells [8]. This raises the question, if restoration of the kidney function after an episode of acute kidney injury (AKI) as frequently observed clinically is a result from sufficiently replaced tubule epithelial cells, or rather from hypertrophy of the cells and hyperfiltration of the remaining nephrons. Consequently, this emphasized the importance of saving remnant tubules after an episode of ATN. Epidemiological data indicate, that the severity of ATN is a strong risk factor for the transition to CKD [2, 3]. However, the mechanism of a certain extent of necrotic injury tipping the balance from “restitutio ad integrum” to insufficient restoration and even further decline of kidney function in long term is unknown.

We have shown, that renal inflammation following ATN plays a central role in the early injury phase. This form of inflammation, induced by damage-associated molecular patterns (DAMPs) released from necrotic tubule cells mediates regulated necrosis of surviving renal parenchymal cells in an auto-amplifying loop [9]. As a consequence of this concept of “necroinflammation” we hypothesized that the extent of inflammation after ATN depends on the extent of necrotic injury. This inflammation however might cause further injury of the remnant tubules once a certain extent of injury has been exceeded, which for that reason might be an important mechanism for the transition from ATN to CKD. Therefore, we studied different ischemia times in experimental unilateral ischemia/reperfusion injury (IRI) to identify a threshold extent of ischemic tubule necrosis, which as “point of no return” leads to progressive injury, fibrosis and atrophy. We hypothesized, that this progress is temporarily associated by increased markers of inflammation and results in atrophy of the ischemic kidney.

## Material and methods

### Animal studies

All animal experimental procedures had been approved by “Regierung von Oberbayern” and were performed according to the German Animal Care and Ethics Legislation. Six to eight weeks old male wild type C57BL/6 N mice (Charles River Laboratories, Sulzfeld, Germany) were kept in groups of five in filter top cages under standardized conditions in a 12 h light/dark cycle. They were anesthetized before unilateral renal pedicle clamping for 15, 25, 35, and 45 min with a three compound mixture containing 0.5 mg/kg medetomidine, 5 mg/kg midazolam and 0.05 mg/kg fentanyl to achieve analgesia, amnesia and hypnosis. Mice were kept in a ventilated heating chamber (Octagon 20 Advance, Brinsea Products Ltd., Weston Super Mare, UK) before and after surgery and on a heating pad during surgery to maintain body temperature between 36.5 °C and 38.5 °C. After clamping via flank incision the kidney was placed back inside the abdomen throughout the ischemia time. Online rectal temperature surveillance was performed for every mouse throughout the whole procedure. Fluid loss was compensated by administration of 200 μl 0.9% NaCl intraperitoneally. 2.5 mg/kg atipamezole, 0.5 mg/kg flumazenil and 1.2 mg/kg naloxone were applied to antagonize anesthesia and 0.05 mg/kg buprenorphin was injected subcutaneously for pain control. Depending on the experimental design, mice were sacrifized after 15 min, 30 min, 1 h, 6 h, 12 h, 24 h, 48 h, 3 d, 4 d, 7 d, 10 d, and 5 weeks. The ischemic kidneys were removed, scaled, decapsulated, divided and stored in 4% buffered formalin or frozen in RNAlater (ThermoFisher, Waltham, USA). Plasma creatinine was measured using the Creatinine FS kit (DiaSys Diagnostic Systems, Holzheim, Germany).

### RNA preparation, reverse transcription and quantitative real-time PCR

Pure Link RNA Mini Kit (Thermo Fisher Scientific, Waltham, USA) was used to isolate RNA from total kidneys according to the manufacturer’s instructions. Two microgram of total RNA were reverse transcribed to synthesize cDNA (Thermo Fisher Scientific, Waltham, USA) as described [10]. qRT-PCR was performed using SYBR Green Dye Detection System (Thermo Fisher Scientific, Waltham, USA) and the qRT-PCR amplification and detection instrument Light Cycler 480 (Roche, Basel, Switzerland). The reference transcript for relative quantification was 18 s rRNA, wherefore all data were normalized to 18 s. (Table 1: Primer pairs used in this study).

**Table 1:** Primer pairs used in this study

Gene		Sequence
*18 s*	Forward Reverse	5′GCAATTATTCCCCATGAA3′ 3′AGGGCCTCACTAAACCAT5′
*Clusterin*	Forward Reverse	5′AAAAGCCGTGCGGAATGAGA3′ 3′TCGCAAGGCGGCTTTTATTG5′
*Col1a1*	Forward Reverse	5′ ACATGTTCAGCTTTGTGGACC3′ 3′TAGGCCATTGTGTATGCAGC5′
*Ccl2*	Forward Reverse	5′ CCTGCTGTTCACAGTTGCC3′ 3′ATTGGGATCATCTTGCTGGT5′
*Il-6*	Forward Reverse	5′ CGGCCTTCCCTACTTCAC3′ 3′GCCATTGCACAACTCTTTTCTCA5′
*Kim-1*	Forward Reverse	5′ TCAGCTCGGGAATGCACAA3′ 3′TGGTTGCCTTCCGTGTCTCT5′
*Laminin (Lamb2)*	Forward Reverse	5′ CATGTGCTGCCTAAGGATGA3′ 3′TCAGCTTGTAGGAGATGCCA5′
*Ngal*	Forward Reverse	5′ ATGTCACCTCCATCCTGGTCAG3′ 3′GCCACTTGCACATTGTAGCTCTG5′
*Tgf-ß1*	Forward Reverse	5′ GGAGAGCCCTGGATACCAAC3′ 3′CAACCCAGGTCCTTCCTAAA5′
*Tnf-α*	Forward Reverse	5′ AGGGTCTGGGCCATAGAACT3′ 3′ CCACCACGCTCTTCTGTCTA5′

*18 s* 18 s ribosomal RNA, *Col1a1* Collagen, type I, α 1, *Ccl-2* chemokine (C-C motif) ligand 2, *Il-6* Interleukin 6, *Kim-1* Kidney injury molecule 1, *Ngal* Neutrophil gelatinase-associated lipocalin, *Tgf-β1* Transforming growth factor β 1, *Tnf-α* Tumor necrosis factor-α

### Histopathological evaluation

All kidneys were decapsulated and stored in 4% buffered formalin for histological evaluation. They were embedded in paraffin and cut into 2–4 μm sections for periodic acid–Schiff (PAS) staining and immunostaining as described elsewhere [11]. A tubular injury score was determined by a blinded observer analyzing 5 high power fields (50x magnification) and scoring the cast formation, vacuolization, loss of brush border and cellular necrosis between 0 and 10 points. The rat anti-mouse Ly-6B.2 (Bio-Rad, Hercules, USA) antibody was used to immunostain neutrophils and the rat anti-mouse F4/80 Antigen antibody (Bio-Rad, Hercules, USA) to immunostain macrophages before quantification with ImageJ [12] and normalization to whole kidney section area. Dying cells were visualized using terminal deoxynucleotidyl transferase dUTP nick end labeling (TUNEL) staining with the cell death detection kit (Roche, Basel, Switzerland) according to the manufacturer’s protocol. Identification of proximal tubule epithelial cells in the co-staining with TUNEL was performed using biotinylated *Lotus tetragonolobus* lectin (Vector Laboratories, Burlingame, USA).

### Statistical analysis

Mice experiments were performed using groups of 5. Data are shown as means with s.e.m. Comparison was performed using univariate ANOVA or student’s t-test as far as only two groups with normally distributed data were compared. *P*-values less than 0.05 were indicated statistically significant (*p* < 0.05). Statistical association was tested using pearson’s correlation coefficient analysis. Coefficient values between +/− 0.8 and +/− 1 were defined “strong positive/negative correlation”.

## Results

### Injury of tubules and levels of serum creatinine at 24 h and 5 weeks after unilateral ischemia for 15, 25, 35, and 45 min

The severity of injury of tubules upon IRI is determined by the murine race, sex, temperature during ischemia and most importantly the duration of ischemia [10, 13–15]. As first step we titrated the duration of ischemia to find the threshold extent of injury between (partial) recovery and persistent injury in a standardized experimental setting, that had been optimized before [10]. Therefore 6–8 weeks old male C57BL/6 N mice underwent unilateral IRI for 15, 25, 35 and 45 min. We assessed kidney injury analyzing gene expression of Kidney injury molecule 1 (*Kim-1),* Neutrophil gelatinase-associated lipocalin *(Ngal)* and *Clusterin* and the semiquantitative tubular injury score between 0 and 10. KIM-1 is a type 1 membrane protein that is upregulated under different disease condition and known as early and sensitive injury marker [16, 17]. Additionally, it not only marks injury but mediates cell repair, too [18]. NGAL not only serves as early injury marker, but also seems to play a role in the transition from acute to chronic kidney disease [19] and its exogenous application experimentally attenuated IRI in rats [20]. Clusterin is a chaperone-like protein that besides early detection of AKI indicates the reabsorption function of tubules [21] and is associated with renal inflammation and fibrosis after IRI [22].

**Fig. 1:**
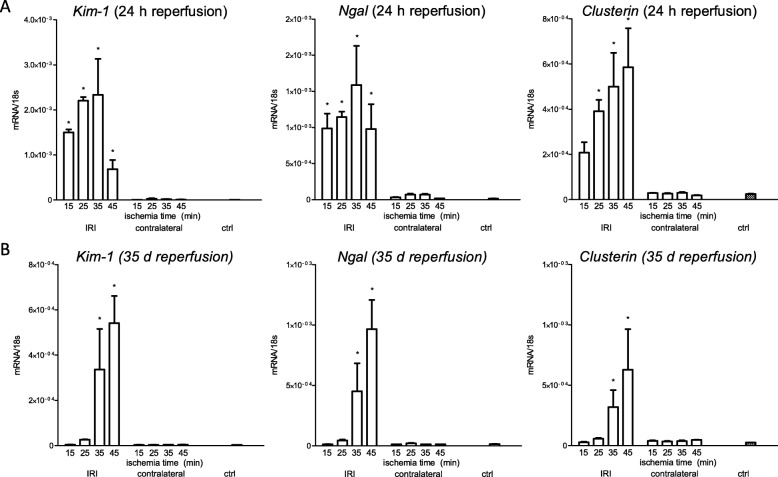
Gene expression of injury markers after ischemia for 15, 25, 35 and 45 min and reperfusion for 24 h and 5 weeks. **a**: 24 h after ischemic injury gene expression of *Kim-1* and *Ngal* were significantly upregulated in any treatment group whereas expression of *Clusterin* was upregulated after 25, 35 and 45 min of ischemia. **b**: The expression of *Kim-1*, *Ngal* and *Clusterin* showed a different pattern following reperfusion of 5 weeks. *Kim*-1, *Ngal* and *Clusterin* were significantly upregulated at the ischemic injury for 35 and 45 min and 5 weeks of reperfusion. Displayed are means with standard deviation *P < 0.05 versus control

**Fig. 2:**
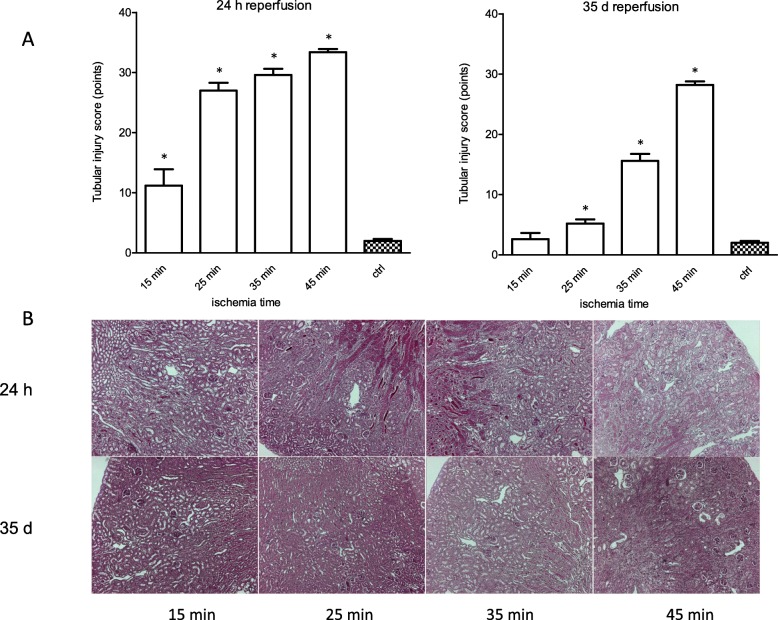
Progressing pathohistological injury with increasing ischemia time after reperfusion for 24 h and 5 weeks. **a**: After 24 h reperfusion any treatment group revealed histological injury, whereas reperfusion for 5 weeks allowed histological healing according to the tubular injury score only in the treatment group of ischemia for 15 min. Unilateral ischemic injury for 25 min, 35 min and 45 min induced persistent (5 weeks) histopathological lesions. **b**: Representative images of PAS stained sections (50x magnification) of renal cortices of kidneys undergone unilateral ischemia for 15 min, 25 min, 35 min and 45 min and reperfusion for 24 h and 5 weeks. Displayed are means with s.e.m. *P < 0.05 versus control

After 24 h reperfusion gene expression of *Kim-1* and *Ngal* showed significant upregulation in any treatment group compared to healthy controls. There was significant upregulation of the gene expression of C*lusterin* following ischemia for 25, 35 and 45 min. At 5 weeks ischemia for 35 and 45 min revealed significant upregulation of the gene expression of *Kim-1*, *Ngal* and *Clusterin*, whereas ischemia for 15 and 25 min did not sustain upregulation 5 weeks after IRI. (Fig. 1) Histologically, significant injury of tubules could be detected following ischemia for 15, 25, 35 and 45 min after 24 h reperfusion whereas only ischemia for 25, 35 and 45 min led to an increased injury of tubules that could still be detected after 5 weeks. (Fig. 2).

Serum creatine was measured to investigate the translation of these changes on a gene expression level into the excretory function of the kidneys. Unilateral IRI for 15, 25, 35 and 45 min did not result in alterations of the serum creatinine after reperfusion for 24 h or 35 d (data not shown). Although not proven we suspect the limitation of serum creatinine as biomarker for alterations in the glomerular filtration rate in the unilateral injury model with compensation of the contralateral kidney as possible reason for the unchanged creatinine values.

### Inflammation at 24 h and 5 weeks after unilateral ischemia for 15, 25, 35, and 45 min

Is there an extent of ischemic injury, which induces upregulation of inflammation markers not only at 24 h but also at 5 weeks? To answer this question, quantification of the gene expression of Tumor necrosis factor-alpha (*Tnf-α*), Chemokine (C-C motif) ligand 2 (*Ccl-2*) and Interleukin 6 (*Il-6*) were performed. TNF-α is a pro-inflammatory cytokine which is released not only by macrophages but also tubule epithelial cells upon injury [23]. Among others it mediates regulated cell death as necroptosis [24], wherefore it is central in the cascade of inflammation and cell death. CCL-2 is a chemokine which attracts monocytes into the tubulointerstitial space after IRI [25] and mediates fibrosis in IgA nephropathy and diabetic nephropathy [26, 27]. As pro-inflammatory cytokine IL-6 mediates systemic and local inflammation in IRI via neutrophil attraction [28, 29].

**Fig. 3:**
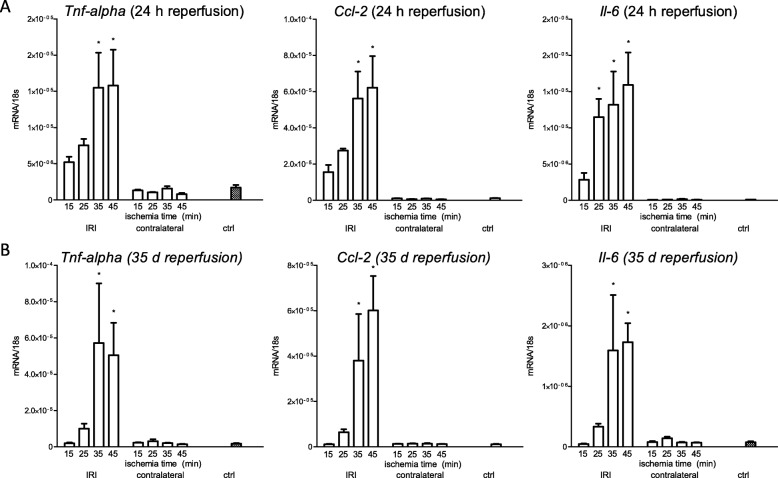
Gene expression of inflammation markers after ischemia for 15, 25, 35 and 45 min and reperfusion for 24 h and 5 weeks. **a**: 24 h after ischemic injury gene expression of *Tnf-α*, *Ccl-2* and *Il-6* were significantly upregulated following ischemia for 35 and 45 min. IL-6 additionally revealed significant gene expression after ischemia for 25 min. **b**: 5 weeks after the ischemic injury the expression of *Tnf-α*, *Ccl-2* and *Il-6* was significantly upregulated only after ischemia for 35 min and 45 min. Displayed are means with s.e.m. *P < 0.05 versus control

Gene expression of *Tnf-α* and *Ccl-2* were significantly upregulated upon 35 and 45 min of ischemia, whereas *Il-6* upregulated after 25, 35 and 45 min of ischemia at 24 h of reperfusion. At 5 weeks however, *Tnf-α*, *Ccl-2* and *Il-6* were upregulated only after 35 and 45 min ischemia time. (Fig. 3).

### Fibrosis at 24 h and 5 weeks after unilateral ischemia for 15, 25, 35, and 45 min

Considering fibrosis only as biomarker for parenchymal loss or as important pathophysiological mechanism of progressing injury is a matter of debate. However, it clearly marks a result of severe injury. Therefore, we investigated gene expression of Transforming growth factor beta 1 (*Tgf-β1*), Collagen, type I, alpha 1 (*Col1a1*) and *Laminin*. TGF-β1 as one member of the TGF-β superfamily was identified as a central mediator in renal fibrosis [30]. It not only functions as (experimental) urinary biomarker in patients with proteinuric kidney disease [31] but also itself mediates synthesis of extracellular matrix and fibroblast proliferation [32, 33]. Chronic kidney disease is characterized by increased extracellular matrix proteins. One major component of this matrix is Collagen I [34], which excessively accumulates in renal fibrosis [35], wherefore the expression of *Col1a1* was crucial to investigate. Laminin is present in the mature glomerular basement membrane under physiological condition [36]. However, the expression of the β chain of Laminin has been observed to be upregulated in the glomerular basement membrane of kidneys of patients with chronic kidney disease of various reasons [37]. Therefore, we chose *Laminin* as representative for thickening of the glomerular basement membrane after IRI.

**Fig. 4:**
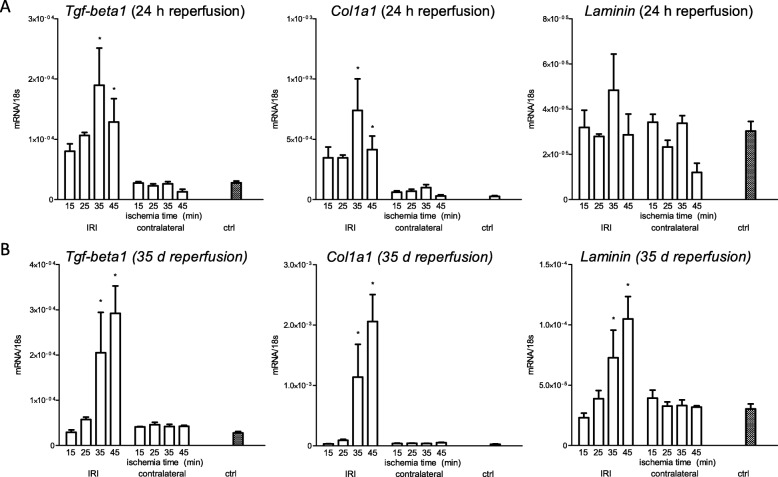
Gene expression of fibrosis markers after ischemia for 15, 25, 35 and 45 min and reperfusion for 24 h and 5 weeks. **a**: 24 h after ischemic injury gene expression of *Tgf-β1* and *Col1a1* significantly upregulated following ischemia for 35 and 45 min, whereas *Laminin* did not reveal significant upregulation in any treatment group following reperfusion for 24 h. **b**: After 5 weeks reperfusion *Tgf-β1*, *Col1a1* and *Laminin* were significantly upregulated in the treatment groups of ischemia for 35 min and 45 min. Displayed are means with s.e.m. *P < 0.05 versus control

*Tgf-β1* and *Col1a1* revealed significant upregulation following 35 and 45 min ischemia after 24 h reperfusion. At 5 weeks and ischemia times of 35 and 45 min the expression of *Tgf-β1*, *Col1a1* and *laminin* were significantly upregulated. (Fig. 4).

Together, 35 min of ischemia time marks a threshold extent of necrotic injury which induces significant upregulation of markers for injury, inflammation and fibrosis not only at 24 h but also at 5 weeks.

### Course of markers for injury and inflammation and correlation analysis after IRI for 35 min

In a second phase of this study we concisely characterized the course of markers for injury and inflammation over max. Five weeks to study the temporal association between necrosis and inflammation as hypothesized in the concept of necroinflammation.

**Fig. 5:**
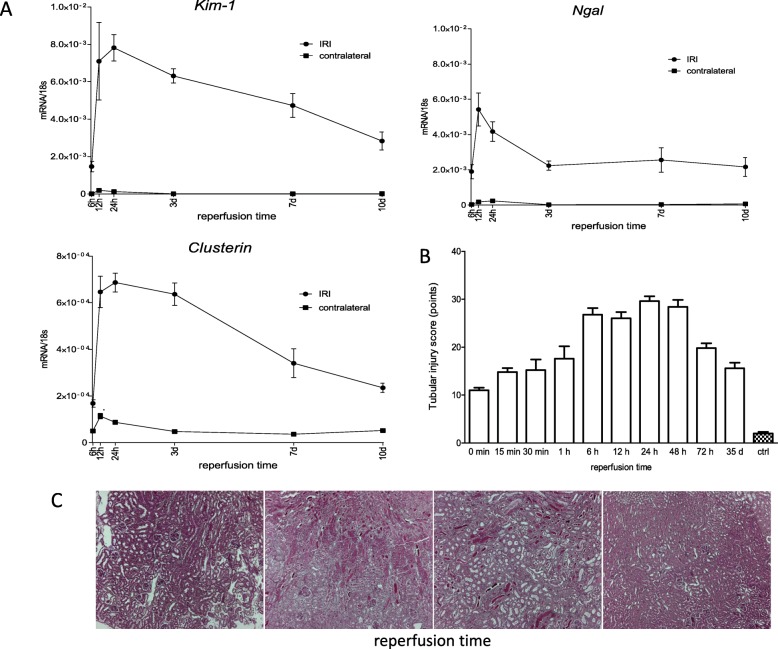
Course of indicators for kidney injury after ischemia for 35 min over different reperfusion times until 5 weeks. **a**: Quantification of expression of the injury markers *Kim-1* and *Clusterin* indicated highest expression between 6 h and 24 h with a constant decline over the period of observation (until day 10), whereas *Ngal* displayed expression on equal level between 3 and 10 days after peaking between 6 h and 24 h. **b**: Histological investigation of the injury of tubules over a period of 5 weeks showed increasing injury until 6 h after the ischemic injury with a plateau until 48 h after injury and decline until the end of observation. **c**: Representative images of PAS stained sections (50x magnification) of renal cortices of kidneys undergone unilateral ischemia for 35 min and multiple reperfusion times between 0 min and 5 weeks. Displayed are means with s.e.m

**Fig. 6:**
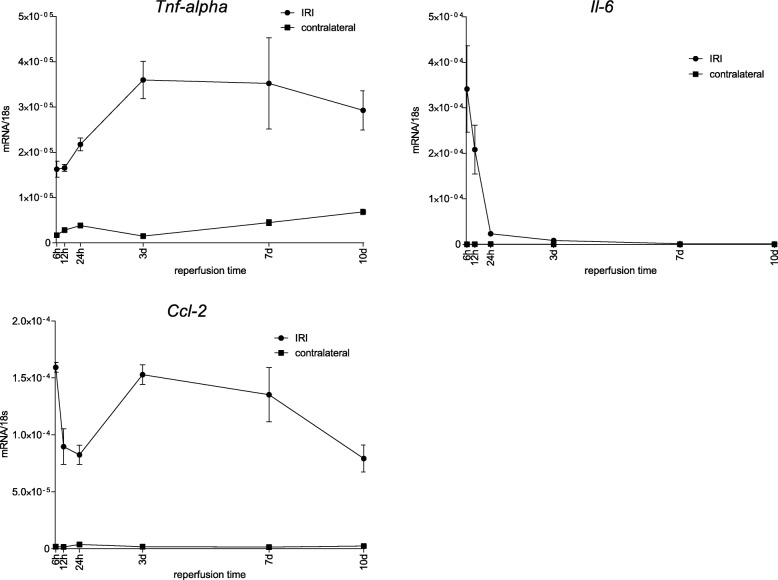
Course of inflammation markers after ischemia for 35 min and reperfusion times between 6 h and 10 d. Quantification of the expression of markers of inflammation showed very different expression patterns. The expression of *Tnf-α* rose between 6 h and 3 d to remain on an equally elevated level throughout the whole period of observation. The expression of *Il-6* peaked at the first time point of observation to a rapid decline, whereas *Ccl-2* started with a high expression at 6 h, dropped to a lower level until 24 h and went back to the elevated level of expression at 3 d to gradually decline until the end of observation period at 10 d. Displayed are means with s.e.m

Along the course of 10 d *Kim-1* and *Clusterin* peaked its gene expression between 6 h and 24 h with a constant decline thereafter, while *Ngal* also peaked between 6 h and 24 h but remained upregulated on a constant level between 3 d and 10 d. (Fig. 5) We investigated the phases of inflammation after the ischemic injury of 35 min ischemia time in an equal experimental setting. Whilst *Tnf-α* solely revealed highest expression between 3 d and 10 d, *Ccl-2* was upregulated after 6 h with a drop until 24 h and again upregulated between 3 d and 7 d. This finding had been reported before and results from the technical limitation of whole kidney expression analysis in this study [38]. *Il-6* peaked its upregulation after 6 h (however, 6 h marked the first time point of investigation, wherefore its upregulation possibly peaked even before) with a rapid drop thereafter. (Fig. 6) Table 2 illustrates features of the time course of all markers for inflammation, injury and fibrosis. While all markers of inflammation and injury showed an onset of significant increase before the first time point of investigation, they peaked at various reperfusion times between 6 h and 3 d. Consequently, the onset of significant decline differed starting from 12 h for Il-6 until 7 d for Ccl-2. Consistently, all markers of fibrosis peaked at 3 d and started to significantly decline between 3 d and 7 d.

**Table 2:**
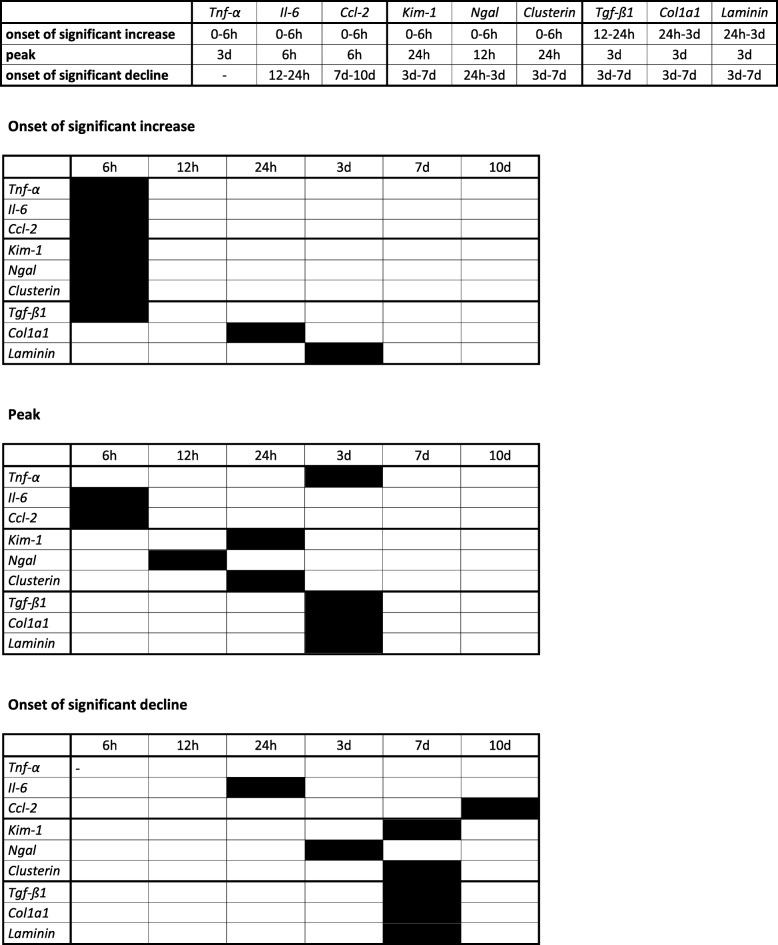
Onset of significant increase, peak, and onset of significant decline along the course of 10 d of gene expression markers for inflammation, injury and fibrosis

*Col1a1* Collagen, type I, α 1, *Ccl-2* chemokine (C-C motif) ligand 2, *Il-6* Interleukin 6, *Kim-1* Kidney injury molecule 1, *Ngal* Neutrophil gelatinase-associated lipocalin, *Tgf-β1* Transforming growth factor β 1, *Tnf-α* Tumor necrosis factor-α

As the gene expression of all markers of injury and inflammation peaked within the first 3 d, we chose to more concisely investigate this time frame with additional focus on the injury of tubules after long term reperfusion for 5 weeks. Assessing the injury of tubules histologically, we found a peak of the injury of tubules between 6 h and 48 h followed by a decline until the end of observation. (Fig. 5).

**Fig. 7:**
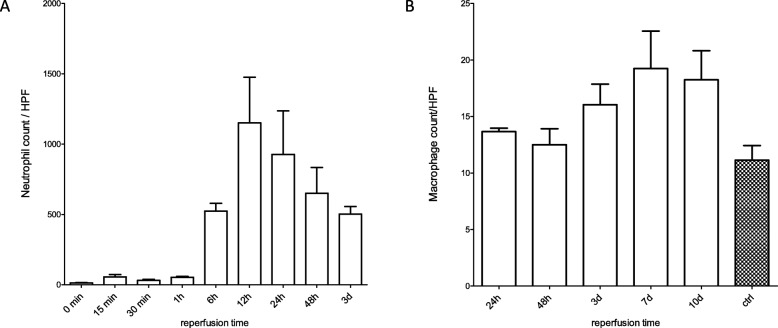
Influx of neutrophils and course of macrophages following ischemia for 35 min at multiple reperfusion times. **a**: The influx of neutrophils peaked at 12 h with a constant decline of intensity of Ly-6B.2 staining until the end of observation period a 3 d. **b**: The macrophage count remained on the same level as the control group until a significant rise at 3 d. Highest macrophage number was counted at 7 d remaining on this level until the end of observation (day 10 d). Displayed are means with s.e.m

Neutrophils function as “first responders” in sterile necrosis of tubules [39]. Therapeutic depletion of neutrophil influx in different models of AKI showed conflicting results, which was partially argued with different severities of the injury of tubules in these different studies as possible reason [40–42]. In our descriptive study neutrophil staining (Ly-6B.2) revealed an elevated influx already after 15 min reperfusion. We detected the peak intensity of staining after 12 h with a subsequent decline. (Fig. 7a).

Macrophages play a key role in renal injury and repair. Depending on the phenotype of the macrophages determined by the renal microenvironment, they have been identified with pro-inflammatory, pro-regenerative, fibrolytic and pro-fibrotic features [43–45]. After IRI the number of macrophages remains on the same level as the control group for 48 h. At 3 d the number of macrophages rises and remains on a peaking level throughout the whole period of investigation most likely as a consequence of monocyte influx and differentiation towards macrophages [46]. (Fig. 7b).

We used the calculation of Pearson’s correlation coefficient to analyze the correlation of the courses of all gene expression markers. Additionally, the correlation of the courses of the tubular injury score and the neutrophil influx was tested and revealed a strong positive correlation. *Tnf-α* and *Il-6 *showed a strong negative correlation, while *Kim-1* and *Clusterin*, *Tnf-α* and *Col1a1*, *Tgf-ß1* and * Col1a1*, *Tgf-ß1* and *Laminin*, and *Col1a1* and *Laminin* showed a strong positive correlation. (Table 3).

**Table 3:**
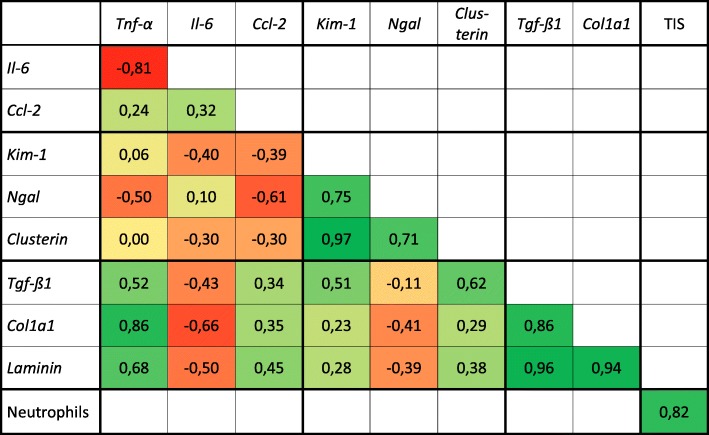
Correlation between gene expression markers for inflammation, injury and fibrosis and between the tubular injury score and the neutrophil influx

Calculation of Pearson’s correlation coefficient indicates the relationship between the gene expression of markers for inflammation, injury and fibrosis along a time course between 6 h and 10 d. The time courses of the tubular injury score and neutrophil influx between 0 h and 3 d was calculated separately. While *Tnf-α* and *Il-6* revealed a strong negative relation, *Kim-1* and *Clusterin*, *Tnf-α* and *Col1a1*, *Tgf-ß1* and *Col1a1*, *Tgf-ß1* and *Laminin*, and *Col1a1* and *Laminin* showed a strong positive correlation. The correlation of the course of the tubular injury score and the neutrophil influx was strong positiv. A correlation coefficient between +/− 0.8 and +/− 1 was defined as „strong positive/negative correlation”

*Col1a1* Collagen, type I, α 1, *Ccl-2* chemokine (C-C motif) ligand 2, *Il-6* Interleukin 6, *Kim-1* Kidney injury molecule 1, *Ngal* Neutrophil gelatinase-associated lipocalin, *Tgf-β1* Transforming growth factor β 1, *Tnf-α* Tumor necrosis factor-α, *TIS* Tubular injury score

### Characterization of cell death

**Fig. 8:**
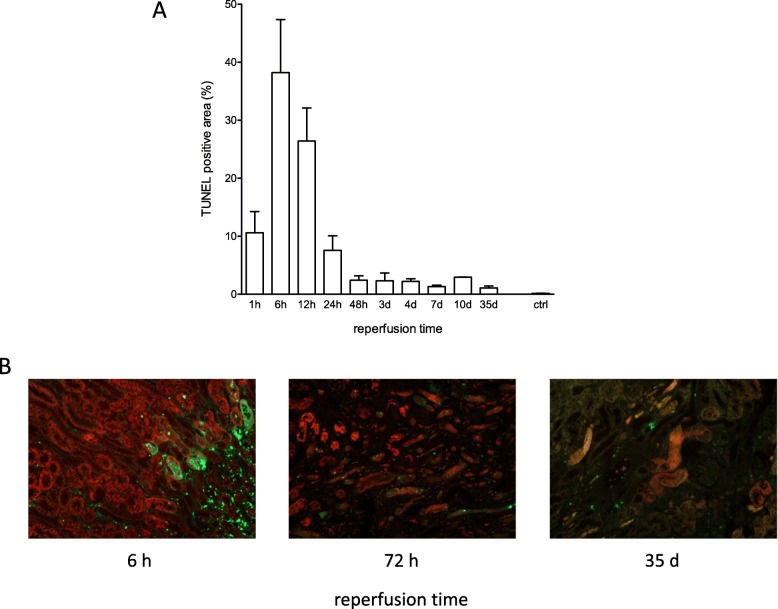
Course of cell death after ischemia for 35 min and reperfusion times between 1 h and 5 weeks. **a**: TUNEL staining of ischemic kidney sections after ischemia for 35 min and assessment at different time points until 5 weeks revealed highest intensity after 6 h with rapid decline to a constant level between 48 h and 35 d. **b**: Images of *Lotus tetragonolobus* lectin (red) and TUNEL (green) co-stained sections (100x magnification) of renal cortices of kidneys undergone unilateral ischemia for 35 min at different reperfusion times. The weakening signal for *Lotus tetragonolobus* lectin indicates progressing tubule atrophy over time and does not allow to define the dying cell type after 72 h and 35 d, whereas the majority of TUNEL positivity derives from proximal tubule epithelial cells after 6 h. Displayed are means of 3 sections analyzed using ImageJ with s.e.m

Characterization of unspecific cell death marked by TUNEL staining revealed highest positivity after 6 h reperfusion with a constant decline to a level, that remained for the whole duration of study (until 5 weeks). Co-staining of TUNEL and lectin confirmed the major source of dying cells to origin from proximal tubule epithelial cells at an early time point of 6 h. However, already after 6 h there was additional TUNEL positivity detected in the interstitial space. As a consequence of progressing kidney atrophy the co-staining with lectin disappeared at late time points wherefore after 5 weeks this co-staining did not allow to define the dying cell type. However, it is important to remark, that the rate of cells dying remains elevated for a period of 5 weeks after acute injury. (Fig. 8).

### Characterization of fibrosis / kidney atrophy

**Fig. 9:**
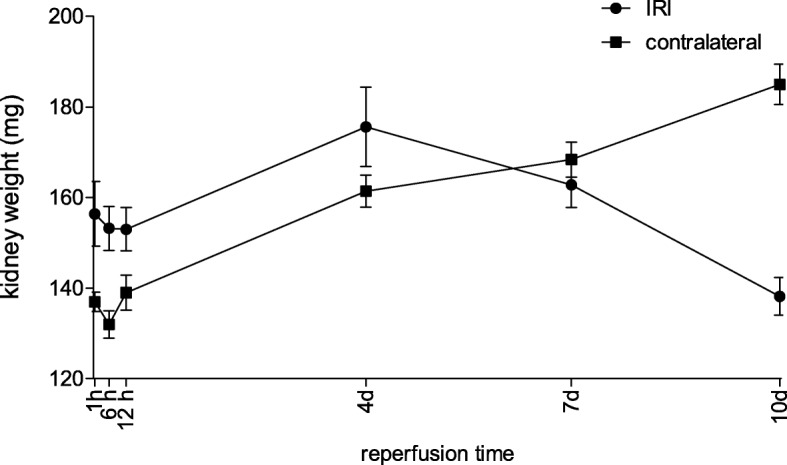
Kidney weight of the ischemic and contralateral kidneys after ischemia for 35 min and reperfusion between 1 h and 10 d. Throughout the period of observation between 1 h and 10 d both the weight of the ischemic and the contralateral kidneys rose from 1 h to 4 d with higher weight of the ischemic kidneys. Between 4 d and 7 d the weight of the ischemic kidneys started to decline until 10 d, whereas the weight of the contralateral kidney rose. Displayed are means with s.e.m

As strongest “structural” predictor for “functional” outcome we assessed the course of the kidney weights between 1 h and 10 d. With ongoing loss of function of the ischemic kidney, there is concomitant hypertrophy of the contralateral kidney. However, initially the ischemic kidneys presented higher weights most likely marking ongoing edema within these kidneys. The inverting point from which the ischemic kidney was lighter than the contralateral kidney was between 4 and 7 d. (Fig. 9).

**Fig. 10:**
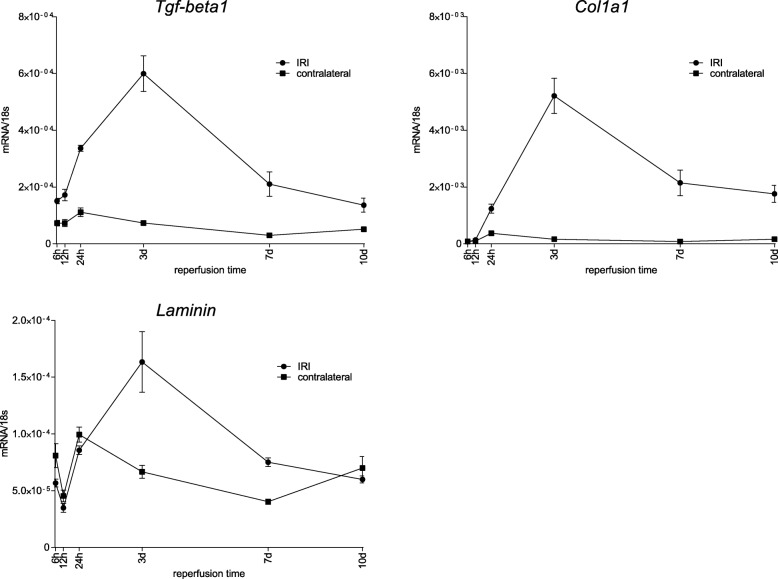
Course of fibrosis marker after ischemia for 35 min over reperfusion times between 6 h and 10 d. The expression of the markers *Tgf-β1*, *Col1a1* and *Laminin* showed very similar patterns of expression. In the ischemic kidneys all markers upregulated their expression until 3 d, followed by a constant decline with remaining expression of *Tgf-β1* and *Col1a1* on an elevated level and decline to baseline expression of *Laminin*. The contralateral kidneys revealed upregulated expression of *Tgf-β1* and *Col1a1*, whereas *Laminin* showed an initial drop of expression at 12 h with a rise at the end of observation period a 10 d. Displayed are means with s.e.m

Ongoing fibrosis was investigated by the gene expression of *Tgf-β1*, *Col1a1* and *Laminin*. These markers consistently showed highest upregulation after 3 d reperfusion followed by a decline. *Tgf-β1* and *Col1a1* still were upregulated at the end of these examinations (10 d reperfusion) whereas laminin downregulated its expression to normal values. (Fig. 10).

## Discussion

Our results indicate 35 min as “point of no return” ischemia time in this experimental setting after unilateral IRI in long term investigation. The injury following ischemia for 35 and 45 min induces persistent upregulation of markers for inflammation, injury, cell death and fibrosis leading to atrophy of the ischemic kidney and compensatory hypertrophy of the contralateral kidney. Additionally, the extent of injury after ischemia for 35 min allows discrimination of improvement but also worsening of the outcome (e.g. in case of intervention studies).

The concise characterization of the course of markers after 35 min unilateral IRI revealed different phases of inflammation, cell death, injury and fibrosis/atrophy. Depending on the marker, this study identified overlaps of their courses throughout the period of investigation. The early incidence of necrotic cell death was associated with the early incidence of inflammation supporting the concept of necroinflammation [47]. The incidence of markers of fibrosis as sign of irreversible loss of functional kidney parenchyma occurred later but progressed to atrophy of the ischemic kidney with compensatory hypertrophy of the contralateral kidney.

Ischemia for only 15 min induced detectably elevated injury and inflammation of the kidney at 24 h. However, after 5 weeks reperfusion there was only upregulation of inflammation, injury, cell death or fibrosis after at least 35 min of ischemia, consistently. Atrophy of the injured kidney with compensating hypertrophy of the contralateral kidney doubtless marks insufficiency of structural repair. Our results indicated an ischemia time of 35 min as threshold ischemia dose for induction of fibrosis and progressing atrophy as 25 min ischemia led to no significant delta kidney weight between the ischemic and contralateral kidney whereas 45 min ischemia exceeded the result.

Together, 35 min of ischemia set the threshold of ischemia for sustained inflammation, injury and fibrosis. The consistency of progressing inflammation/injury and fibrosis/atrophy after this threshold dose of 35 min is remarkable keeping in mind that in this long term study the injurious trigger had been terminated 5 weeks ago. This suggests a pathophysiological mechanism, which might trigger itself in a vicious circle once a threshold of injurious trigger has been exceeded. The underlying mechanism between an elevated state of inflammation and the perpetuation of injury is unclear.

The simultaneous upregulation of markers for inflammation and injury/cell death might interlink in a mechanism of necroinflammation in which inflammation and necrosis condition and enforce each other [48]. In our setting this pathophysiological mechanism starts with cell death and a consequent release of DAMPs. These DAMPs induce a pro-inflammatory environment which activate regulated forms of cell death like necroptosis, pyroptosis, mitochondrial-permeability transition-mediated regulated necrosis and ferroptosis in surrounding tubule epithelial cells which in turn release more DAMPs and facilitate this vicious circle [47]. Senescence of tubule epithelial cells has been studied as another pathophysiological interconnection between inflammation and the development of CKD. Cell cycle arrest (as definition of senescence) in tubule epithelial cells causes renal fibrosis via the TLR/IL-1R pathway. However, inactivation of this pathway in myeloid differentiation (Myd88) knockout mice consequently reduced renal fibrosis but not damage of tubules after AKI [49].

Investigation of alterations in inflammation, injury, cell death and fibrosis over a course of 10 d reveals two phases. The first phase is characterized as phase in which the markers for inflammation, injury and cell death peak their upregulation. This phase marks the immediate response to the original injurious trigger and is seen within the first 3 days in our experimental setting. In a second phase the markers for inflammation, injury and cell death decrease to a level of consolidation in which they remain throughout the whole observational period. Gene expression of markers of fibrosis peak in this phase beginning 3 days after the ischemic injury. Additionally, progressing atrophy and compensatory hypertrophy of the contralateral kidney characterize the second phase, in which an auto-amplifying loop of necroinflammation might be the driving force of ongoing inflammation, injury and cell death.

As stated above, the mechanism tipping the balance from adaptive repair to loss of function and atrophy after a critical level of injury has been exceeded is not clear. The elevated state of inflammation might serve as important pathophysiological mechanism in post-AKI renal scaring after the excess of a certain level of injurious trigger. Together with the onset of (regulated) cell death many days after the ischemic injury as in the concept of necroinflammation this form of inflammation might serve as potential therapeutic targets.

The identification of a prolonged injury phase is clinically highly relevant as most patients are medically seen after the onset of ATN as after cardiac arrest or hemodynamic shock. Further studies are needed not only to contribute to clear definitions of the different phases from the episode of ATN towards CKD, but also to screen interventions applied at a delayed phase in order to prolong the window of treatment. The results of this study suggest cell death inhibitors or suppressors of inflammation to be tested in future studies. Thereby this study serves as early step to help pave the way for a delayed, phase dependent and precise therapy of ATN to avoid transition to CKD [50].

## Conclusions

In this study we identified a threshold extent of ischemic injury where markers of injury, inflammation and fibrosis remain upregulated for 5 weeks eventually leading to persistent cell death and progressing atrophy. Particularly, the upregulated markers of inflammation and ongoing cell death suggest a progressing process, which is activated only once a certain extent of ischemic injury has been exceeded. This raises hope for the ability to therapeutically intervene on this process, even many hours or days after onset of the ischemic injury to beneficially influence the outcome of chronic on acute kidney injury.

## Data Availability

The datasets used and/or analysed during the current study are available from the corresponding author on reasonable request.

## Abbreviations

AKI: Acute kidney injury
ANOVA: Analysis of variance
ATN: Acute tubule necrosis
CCL-2: Chemokine (C-C motif) ligand 2
CKD: Chronic kidney disease
Col1α1: Collagen, type I, alpha 1
DAMPs: Damage-associated molecular patterns
IL-6: Interleukin 6
IRI: Ischemia/reperfusion injury
KIM-1: Kidney injury molecule 1
NGAL: Neutrophil gelatinase-associated lipocalin
PAS: Periodic acid–Schiff stain
PCR: Polymerase chain reaction
TGF-β1: Transforming growth factor beta 1
TNF-α: Tumor necrosis factor-alpha
TUNEL: Terminal deoxynucleotidyl transferase dUTP nick end labeling
